# Study on the Combined Effects and Mechanisms of Acupoint Catgut Embedding in Improving Sleep Quality and Controlling Asthma Symptoms in Patients With Asthma

**DOI:** 10.1111/crj.70091

**Published:** 2025-07-03

**Authors:** Zhihong Shi, Wulanqiqige, Qing Quan, Narentuya Zhao, Yanan Wang, Xiaohong Bai, Xiaoyan Hu

**Affiliations:** ^1^ Mongolian Medical Department Affiliated Hospital of Inner Mongolia Medical University Hohhot China; ^2^ Mongolian Medicine College Inner Mongolia Medical University Hohhot China; ^3^ Traditional Orthopedics Affiliated Hospital of Inner Mongolia University for Nationalities TongTiao China; ^4^ Department of Psychosomatic Medicine Xilin Gol League Anshen Hospital Xilinhot China; ^5^ Imaging Department Xilingol League Mongolian Medical Hospital Xilinhot China; ^6^ Brain Disease Department Inner Mongolia International Mongolian Medical Hospital Hohhot China

**Keywords:** acupoint catgut embedding therapy, asthma control, sleep quality

## Abstract

**Background:**

Asthma is a chronic airway inflammatory disease that significantly affects patients' quality of life and mental health. This study aims to compare the differences between acupoint embedding auxiliary therapy and traditional auxiliary therapy in asthma control, immune response, sleep quality, and quality of life, as well as evaluate their therapeutic effects on patients with asthma.

**Methods:**

A retrospective analysis of 300 patients with asthma was conducted, divided into acupoint catgut embedding and conventional treatment groups. Key assessments included asthma control (ACQ score), exacerbation frequency, HADS, PSQI, and SF‐36 for quality of life. Immune markers (IgE, eosinophil count, IL‐4, IL‐5, IL‐10) were measured. Univariate and multivariate logistic regression analyses and ROC curves were used to identify predictors of asthma control and sleep quality. Stratified analysis evaluated differences by asthma severity.

**Results:**

The acupoint catgut embedding group showed significant improvements in asthma control (*p* < 0.001), exacerbation frequency (*p* < 0.05), and anxiety and depression (*p* = 0.0359) compared to the conventional treatment group. IgE (*p* = 0.00656), eosinophil count (*p* = 0.0214), and IL‐5 (*p* = 0.0187) were significantly lower, while IL‐10 (*p* = 0.0226) was higher in the acupoint group. Sleep quality (PSQI score, *p* = 0.0117) and deep sleep (NREM 3, *p* < 0.05) improved. Asthma severity (*p* < 0.001) and treatment method (*p* < 0.001) were significant predictors of outcomes, with model 2 (AUC = 0.731) outperforming model 1 (AUC = 0.649). Stratified analysis showed acupoint therapy was more effective in intermittent and mild asthma.

**Conclusion:**

As an auxiliary treatment, acupoint embedding therapy has shown a good effect in improving the sleep quality of patients with asthma and controlling asthma symptoms, especially in patients with mild‐to‐moderate asthma.

## Graphical abstract

The analysis of the effects of acupoint catgut embedding therapy on asthma control, sleep quality, as well as immune and inflammatory factors. Univariate and multivariate analysis of the effects of acupoint catgut embedding therapy.

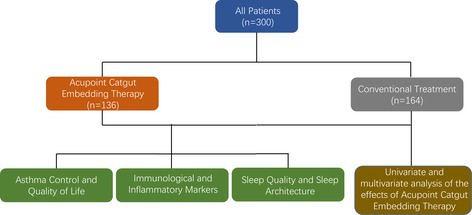

## 1 Introduction

Asthma, as one of the most common chronic respiratory diseases worldwide, seriously affects the quality of life of patients [1, 2]. There are about 300 million patients with asthma worldwide, and the incidence rate continues to rise. The pathological characteristics of asthma include chronic airway inflammation, airway hyperresponsiveness, and reversible airflow limitation, which often lead to symptoms such as shortness of breath, wheezing, coughing, and chest tightness in patients [3]. Long‐term poor control of asthma symptoms not only increases the medical burden on patients, but may also lead to complications such as chronic obstructive pulmonary disease (COPD), which can worsen the condition [4, 5]. Therefore, how to optimize the treatment plan for asthma and improve disease control rate is an important challenge in current clinical management.

At present, the main treatment methods for asthma include drug therapy (such as inhaled corticosteroids [ICS], long‐acting beta 2 receptor agonists, and short acting beta 2 receptor agonists) [6]. These methods have significant effects in improving patient symptoms and reducing the risk of acute attacks [7]. However, some patients still suffer from poor symptom control after receiving standard drug treatment, such as persistent nighttime symptoms and decreased sleep quality. In addition, long‐term drug use may bring certain side effects [8]. Therefore, it is of great significance to find effective adjuvant treatment methods to further optimize asthma management. In recent years, traditional Chinese medicine acupoint embedding therapy has gradually received attention. As a non‐invasive and low side effect treatment method, acupoint embedding therapy continuously stimulates specific acupoints, regulates meridians, qi, and blood, thereby improving airway function and reducing inflammatory reactions [9, 10].

Previous studies have shown that acupoint embedding therapy has certain therapeutic effects in alleviating asthma symptoms and may alleviate airway inflammation through immune regulatory mechanisms [11]. Acupoint catgut embedding therapy generally produces certain therapeutic effects within approximately 2 weeks. However, the exact onset time may vary depending on individual differences, disease severity, and other factors. However, there is still a lack of systematic research on the specific mechanisms of acupoint embedding in improving asthma control (such as ACQ scores on asthma control questionnaires), reducing the frequency of acute attacks, and regulating patients' psychological states (such as anxiety and depression levels). In addition, patients with asthma generally have sleep disorders such as difficulty falling asleep, frequent awakenings, poor sleep quality, etc., which are closely related to the severity of asthma in patients [12]. Sleep disorders not only affect patients' quality of life, but may also exacerbate asthma attacks through complex neuroendocrine regulatory mechanisms [13]. Disruptions in sleep structure, such as reduced rapid eye movement (REM) sleep or insufficient deep non‐rapid eye movement (NREM 3) sleep, may affect patients' airway responsiveness and systemic inflammatory response [14].

This study aims to evaluate the effects of acupoint embedding therapy on asthma control, immune response, sleep quality, and psychological status by comparing it with traditional treatment methods. Through stratified analysis of patients with different degrees of asthma severity, we hope to explore whether acupoint embedding therapy has a more significant advantage in improving the efficacy of patients with mild‐to‐moderate asthma. Our research goal is to provide new evidence for the comprehensive treatment of asthma, verify the clinical application potential of acupoint embedding therapy, especially in improving asthma control rates and improving patient sleep quality.

## 2 Materials and Methods

### 2.1 Study Design and Participants

This retrospective controlled study included patients with asthma treated at our hospital's respiratory department between January 2020 and January 2023. All patients met the criteria of the Global Initiative for Asthma (GINA) and were classified based on asthma severity into intermittent asthma, mild persistent asthma, moderate persistent asthma, and severe persistent asthma. Exclusion criteria included severe systemic diseases (e.g., heart, lung, liver, kidney), recent acute respiratory infections or other complications that could interfere with results, pregnant or breastfeeding women, and patients who were non‐compliant or refused to continue participating in the study. A total of 300 patients were enrolled and divided into two groups: the acupoint catgut embedding therapy group (*n* = 136) and the conventional treatment group (*n* = 164).

This study was conducted in accordance with the Helsinki Declaration, and all participants signed a written informed consent form and obtained approval from the ethics committee.

### 2.2 Treatment Methods

Acupoint Embedding Therapy Group: Patients in this group received acupoint embedding therapy, with key respiratory‐related acupoints selected (e.g., Feishu, Dingchuan, Tiantu). During the procedure, medical absorbable catgut (diameter approximately 0.5–0.7 mm) was implanted subcutaneously at the selected acupoints. The treatment was conducted under sterile conditions: after local disinfection, a specialized needle was used to implant the catgut into the acupoints. The embedded catgut provides continuous stimulation to the acupoints, gradually absorbing within the body, stimulating meridians and blood circulation, regulating lung function, enhancing immunity, and improving respiratory function. The treatment lasted for 12 weeks, with one embedding session every 2 weeks (total of six sessions).

Traditional Auxiliary Treatment Group: Patients in this group performed aerobic walking exercises. Each session began with a 5‐min warm‐up involving slow walking and joint movements to adjust their state. The main training phase consisted of moderate‐intensity walking (speed approximately 4–6 km/h) for 15–20 min per session, synchronized with nasal inhalation and oral exhalation. The exercise was carried out continuously for 12 weeks.

All patients in both groups received standard asthma medication treatment, including ICS, long‐acting beta2‐agonists (LABA), and short‐acting beta2‐agonists (SABA). The treatment duration for both groups was 12 weeks.

### 2.3 Data Collection

After 12 weeks of treatment, asthma control, immune response, sleep quality, and psychological state were evaluated. Primary outcome measures included:

Asthma Control: Assessed using the Asthma Control Questionnaire (ACQ), with scores ranging from 0–6 (higher scores indicate worse control) [15].

Frequency of Acute Exacerbations: Recorded as 1–3, 3–6, or more than 6 exacerbations in 12 weeks.

Pulmonary function indicators: Peak Expiratory Flow (PEF), Forced Expiratory Volume in 1 s (FEV1), Forced Expiratory Volume in 1 s to Forced Vital Capacity ratio (FEV1/FVC).

Immune Response: Blood tests were used to measure IgE, eosinophil count, IL‐4, IL‐5, and IL‐10 levels.

Sleep Quality: Assessed using the PSQI (range 0–21, with higher scores indicating worse sleep quality), along with polysomnography to record REM and NREM sleep percentages, awakenings, and sleep latency [16].

Psychological State: Evaluated using the HADS, where higher scores indicate more severe anxiety and depression. Quality of life was assessed using the SF‐36, which covers eight health domains. Higher scores indicate better health status.

Patients whose ACQ scores decreased by 0.5 or more after treatment were considered to have achieved effective asthma control. A reduction of 3 or more points in PSQI indicated effective sleep quality improvement. Effective treatment was defined as both ACQ and PSQI improvement; all other cases were considered ineffective.

### 2.4 Univariate and Multivariate Logistic Regression Analysis

To assess factors influencing asthma control and sleep quality improvement, univariate logistic regression analysis was first performed to screen for significant variables. Multivariate logistic regression was then conducted to adjust for confounders (e.g., age, gender, smoking, alcohol consumption) and evaluate the independent effects of each factor. Two multivariate models were constructed. Model 1: Included family history of asthma, asthma severity, age, gender, smoking, and alcohol consumption to assess the impact of these variables on asthma control and sleep quality. Model 2: Added the auxiliary treatment method (acupoint catgut embedding or conventional treatment) as a key variable, assessing its independent contribution to asthma control and sleep quality improvement.

### 2.5 Statistical Analysis

Data were analyzed using R software. Quantitative data were expressed as median (range) and compared using the Mann–Whitney U test. Qualitative data were presented as frequencies and percentages, with comparisons made using the chi‐square test. *p* < 0.05 was considered statistically significant.

## 3 Results

### 3.1 Baseline Data Differences Between the Two Auxiliary Treatment Methods

The median age of the patients was 33 years, with an age range of 18–49 years. There were 155 males (51.67%) and 145 females (48.33%). Ninety‐nine patients (33%) were smokers. One hundred sixty‐seven patients (55.67%) had a habit of drinking alcohol. One hundred six patients (35.33%) had a family history of asthma. One hundred sixty‐eight patients (56%) had intermittent asthma, 76 patients (25.33%) had mild persistent asthma, 38 patients (12.67%) had moderate persistent asthma, and 18 patients (6%) had severe persistent asthma (Table 1).

**TABLE 1:** Baseline information of patients with asthma under two treatment methods.

	All patients (*n* = 300)	Acupoint catgut embedding therapy (*n* = 136)	Conventional treatment (*n* = 164)	*p*
Age	33 (18–49)	33 (18–49)	33 (18–49)	0.52
Gender				0.1045997
Male	155 (51.67%)	63 (46.32%)	92 (56.1%)	
Female	145 (48.33%)	73 (53.68%)	72 (43.9%)	
Smoking				0.4612596
Yes	99 (33%)	48 (35.29%)	51 (31.1%)	
No	201 (67%)	88 (64.71%)	113 (68.9%)	
Drinking				0.5604312
Yes	167 (55.67%)	73 (53.68%)	94 (57.32%)	
No	133 (44.33%)	63 (46.32%)	70 (42.68%)	
Family history of asthma				0.1178
Yes	106 (35.33%)	55 (40.44%)	51 (31.10%)	
No	194 (64.67%)	81 (59.56%)	113 (68.90%)	
Asthma severity				0.702
Intermittent asthma	168 (56%)	81(59.56%)	87 (53.05%)	
Mild Persistent asthma	76 (25.33%)	32 (23.53%)	44 (26.83%)	
Moderate persistent asthma	38 (12.67%)	15 (11.03%)	23 (14.02%)	
Severe persistent asthma	18 (6%)	8 (5.88%)	10 (6.10%)	

### 3.2 Comparison of Asthma Control and Quality of Life Between Acupoint Catgut Embedding and Conventional Treatment

After treatment, the acupoint catgut embedding group demonstrated significantly better outcomes in terms of asthma control (ACQ score, *p* < 0.001), frequency of acute exacerbations (*p* < 0.05), and anxiety and depression levels (HADS score, *p* = 0.0359) compared to the conventional treatment group. However, there was no significant difference between the two groups in quality of life scores (SF‐36, *p* = 0.211). The pulmonary function indicators (PEF, FEV1, FEV1/FVC) of the acupoint catgut embedding therapy group were significantly higher than those of the conventional treatment group (*p* = 0.00103, *p* = 0.00483, *p* = 0.000901). One hundred and sixty patients were determined to have effective treatment and 140 patients were determined to have ineffective treatment, with a total treatment effectiveness rate of 53.33%. The treatment effectiveness rate of the acupoint embedding method was 66.18%, while that of the traditional treatment method was 42.68%. The therapeutic effect of acupoint embedding therapy is significantly higher than that of traditional treatment methods (*p* < 0.001) (Table 2).

**TABLE 2:** Comparison of asthma control and quality of life between acupoint catgut embedding therapy and conventional treatment.

	All patients (*n* = 300)	Acupoint catgut embedding therapy (*n* = 136)	Conventional treatment (*n* = 164)	*p*
ACQ	1.8 (0.5–5.5)	1.6 (0.5–5.0)	2.0 (1.0–5.5)	0.000708
Frequency of asthma exacerbations in the year following treatment				3.77E‐02
0 times	222 (74%)	105 (77.21%)	117 (71.34%)	
1–2 times	63 (21%)	29 (21.32%)	34 (20.73%)	
More than 3 times	15 (5%)	2 (1.47%)	13 (7.93%)	
SF‐63	57 (10.6–98.6)	60.2 (10.6–98.6)	54.3 (10.7–98.3)	0.211
HADS	8 (1–16)	7 (1–12)	9 (2–16)	0.0359
Treatment effect				0.0000801
Effective	160 (53.33%)	90 (66.18%)	70 (42.68%	
Ineffective	140 (46.67%)	46 (33.82%)	94 (57.32)	
Peak expiratory flow, PEF (L/min)	385 (320–450)	396 (324–450)	377 (320–448)	0.00103
Forced expiratory volume in 1 s, FEV1 (%)	83.0 (76.6–88.8)	83.4 (76.6–88.8)	82.1 (76.6–88.8)	0.00483
Forced expiratory volume in 1 s to forced vital capacity ratio, FEV1/FVC	73.3 (69.0–77.5)	73.9 (69.0–77.5)	72.4 (69.0–77.5)	0.000901

### 3.3 Differences in Immunological and Inflammatory Markers Between Acupoint Catgut Embedding and Conventional Treatment in Patients with Asthma

Results showed that the levels of IgE (*p* = 0.00656), eosinophil count (*p* = 0.0214), and IL‐5 (*p* = 0.0187) were significantly lower in the acupoint catgut embedding group, while the IL‐10 level was significantly higher (*p* = 0.0226) compared to the conventional treatment group. There was no significant difference in IL‐4 levels between the two groups (*p* = 0.658). These findings suggest that acupoint catgut embedding therapy may modulate immune responses differently in patients with asthma (Table 3).

**TABLE 3:** Comparison of immunological and inflammatory markers between acupoint catgut embedding therapy and conventional treatment in patients with asthma.

	All patients (*n* = 300)	Acupoint catgut embedding therapy (*n* = 136)	Conventional treatment (*n* = 164)	*p*
IgE (IU/mL)	402 (88.4–1060)	378 (88.4–695)	440 (92.6–1060)	0.00656
Absolute eosinophil count (cells/μL)	505 (188–1120)	495 (195–993)	520 (188–1120)	0.0214
IL‐4 (pg/mL)	27.7 (4.19–60.6)	27.6 (4.37–58.9)	28.5 (4.19–60.6)	0.658
IL‐5 (pg/mL)	24.4 (1.84–65.4)	21.1 (1.87–57.3)	26.1 (1.84–65.4)	0.0187
IL‐10 (pg/mL)	6.78 (0.42–15.6)	7.27 (0.45–15.6)	6.58 (0.42–12.8)	0.0226

### 3.4 Comparison of Sleep Quality Improvement Between Acupoint Catgut Embedding and Conventional Treatment in Patients with Asthma

The acupoint catgut embedding group had significantly lower PSQI scores (*p* = 0.0117), fewer awakenings (*p* = 0.0181), and significantly higher proportions of REM sleep (*p* = 0.0194) and NREM 3 (deep sleep, *p* = 0.0077). There were no significant differences between the two groups in sleep latency (*p* = 0.152) and NREM 1 stage (*p* = 0.736), but the difference in NREM 2 stage was statistically significant (*p* = 0.0289). Overall, acupoint catgut embedding therapy demonstrated advantages in improving sleep quality and sleep structure (Table 4).

**TABLE 4:** Comparison of sleep quality and sleep architecture between acupoint catgut embedding therapy and conventional treatment in patients with asthma.

	All patients (*n* = 300)	Acupoint catgut embedding therapy (*n* = 136)	Conventional treatment (*n* = 164)	*p*
PSQI	10 (3–19)	9 (3–17)	11 (6–19)	0.0117
Number of Awakenings	3.44 (0–7)	1 (0–4)	3 (2–7)	0.0181
Sleep latency (min)	30 (15.2–59.4)	29.5 (15.2–59.4)	31 (19.5–50.7)	0.152
REM (%)	17.2 (9.3–27.4)	19.4 (9.3–25.1)	16.6 (10.6–27.4)	0.0194
NREM 1 (%)	8.08 (3.9–12.4)	8.06 (3.9–12.4)	8.11 (4.2–10.7)	0.736
NREM 2 (%)	43.2 (30.4–54.5)	43.8 (34.5–54.5)	42.9 (30.4–48.7)	0.0289
NREM 3 (%)	13 (6.3–22.8)	14.6 (8–22.8)	12.6 (6.3–19.4)	0.0077

### 3.5 Univariate and Multivariate Logistic Regression Analysis of Factors Affecting Good Asthma Control and Sleep Quality Improvement

Univariate analysis showed that family history of asthma (*p* = 0.011) and asthma severity (*p* < 0.001) had significant negative impacts on asthma control and sleep quality, while treatment method (*p* < 0.001) had a significant positive impact, indicating that acupoint catgut embedding therapy significantly improved asthma control and sleep quality. Age, gender, smoking, and alcohol consumption did not show significant effects (*p* > 0.05). In multivariate model 1, family history of asthma (*p* = 0.021, OR = 0.684) and asthma severity (*p* < 0.001, OR = 0.587) had significant negative impacts on asthma control. Age, gender, smoking, and alcohol consumption did not show significant effects in the multivariate model (*p* > 0.05). In multivariate model 2, asthma severity (*p* < 0.001, OR = 0.609) and treatment method (*p* < 0.001, OR = 3.693) remained significant factors affecting asthma control and sleep quality improvement after adjusting for more variables. Other factors (e.g., age, gender, smoking, alcohol consumption) did not show significant effects (*p* > 0.05). Overall, asthma severity and treatment method were significant factors influencing asthma control and sleep quality, with acupoint catgut embedding therapy having the most significant effect on improvement (Table 5).

**TABLE 5:** Univariate and multivariate logistic regression analysis of factors influencing good asthma control and improved sleep quality.

	Estimate	Std error	Z‐Value	*p*			
Family history of asthma*	−0.403	0.158	−2.556	0.011			
Asthma severity*	−0.526	0.081	−6.461	0.000			
Age	0.008	0.005	1.460	0.144			
Gender	−0.043	0.147	−0.296	0.767			
Smoking	−0.168	0.157	−1.072	0.284			
Drinking	−0.182	0.150	−1.216	0.224			
Treatment*	1.367	0.157	8.719	0.000			
Model 1	Estimate	Std error	Z‐Value	*p* value	OR	CI‐lower	CI‐upper
Family history of asthma*	−0.380	0.164	−2.315	0.021	0.684	0.496	0.943
Asthma severity*	−0.532	0.083	−6.418	0.000	0.587	0.499	0.691
Age	0.009	0.006	1.513	0.130	1.009	0.997	1.020
Gender	−0.130	0.153	−0.846	0.397	0.878	0.650	1.186
Smoking	−0.101	0.164	−0.619	0.536	0.904	0.656	1.246
Drinking	−0.276	0.156	−1.765	0.078	0.759	0.559	1.031
Model 2	Estimate	Std error	Z‐Value	*p* value	OR	CI‐lower	CI‐upper
Family history of asthma	−0.279	0.172	−1.622	0.105	0.756	0.540	1.060
Asthma severity*	−0.496	0.087	−5.728	0.000	0.609	0.514	0.721
Age	0.009	0.006	1.595	0.111	1.009	0.998	1.021
Gender	−0.195	0.161	−1.213	0.225	0.822	0.600	1.128
Smoking	−0.136	0.172	−0.788	0.430	0.873	0.623	1.224
Drinking	−0.148	0.164	−0.901	0.368	0.862	0.625	1.190
Treatment*	1.306	0.163	8.026	0.000	3.693	2.684	5.081

* Indicates significant factors in univariate and multivariate Logistic regression analyses.

### 3.6 ROC Analysis of the Performance of the Two Multivariate Models

**FIGURE 1:**
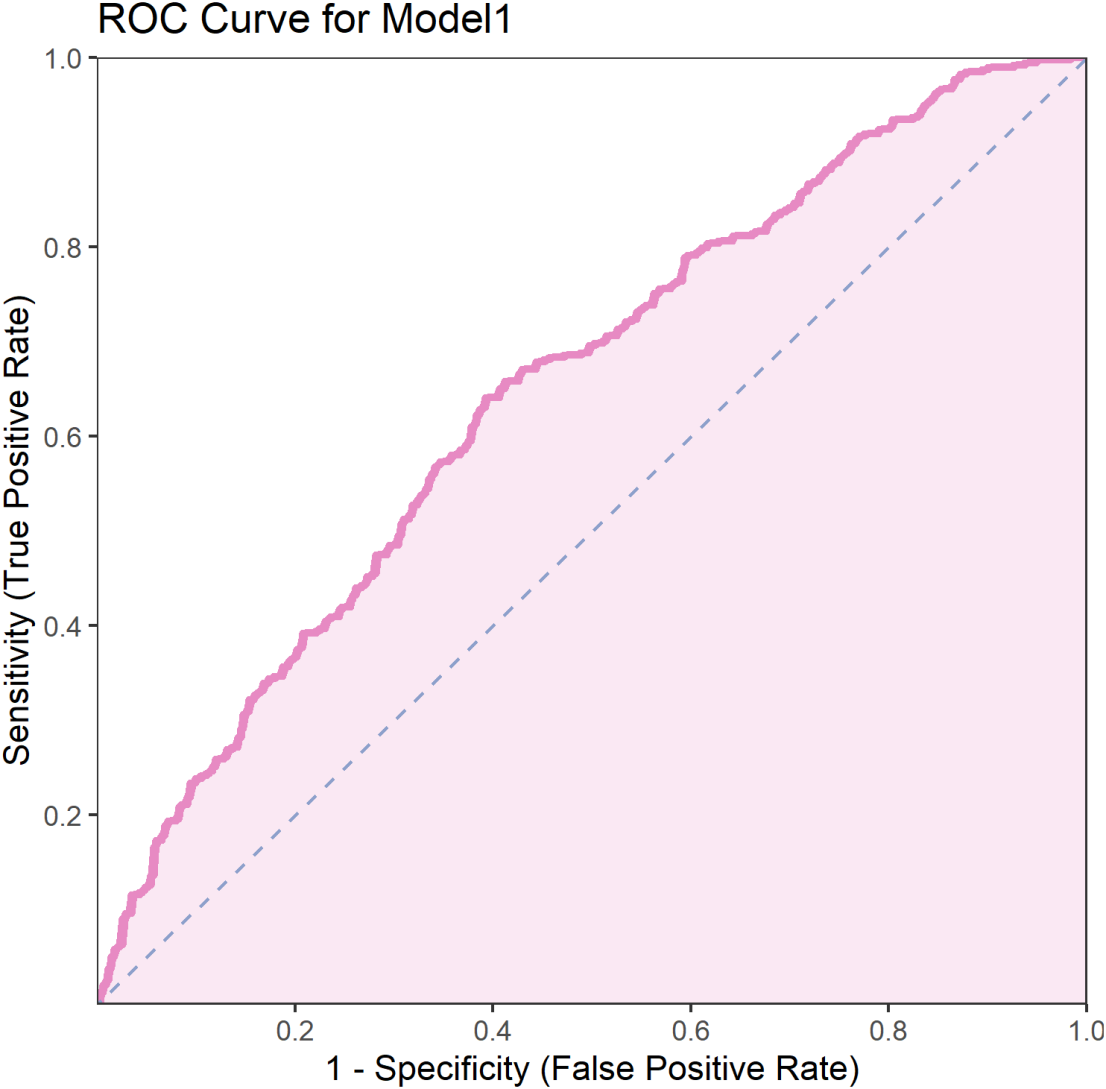
ROC curve for Model 1.

**FIGURE 2:**
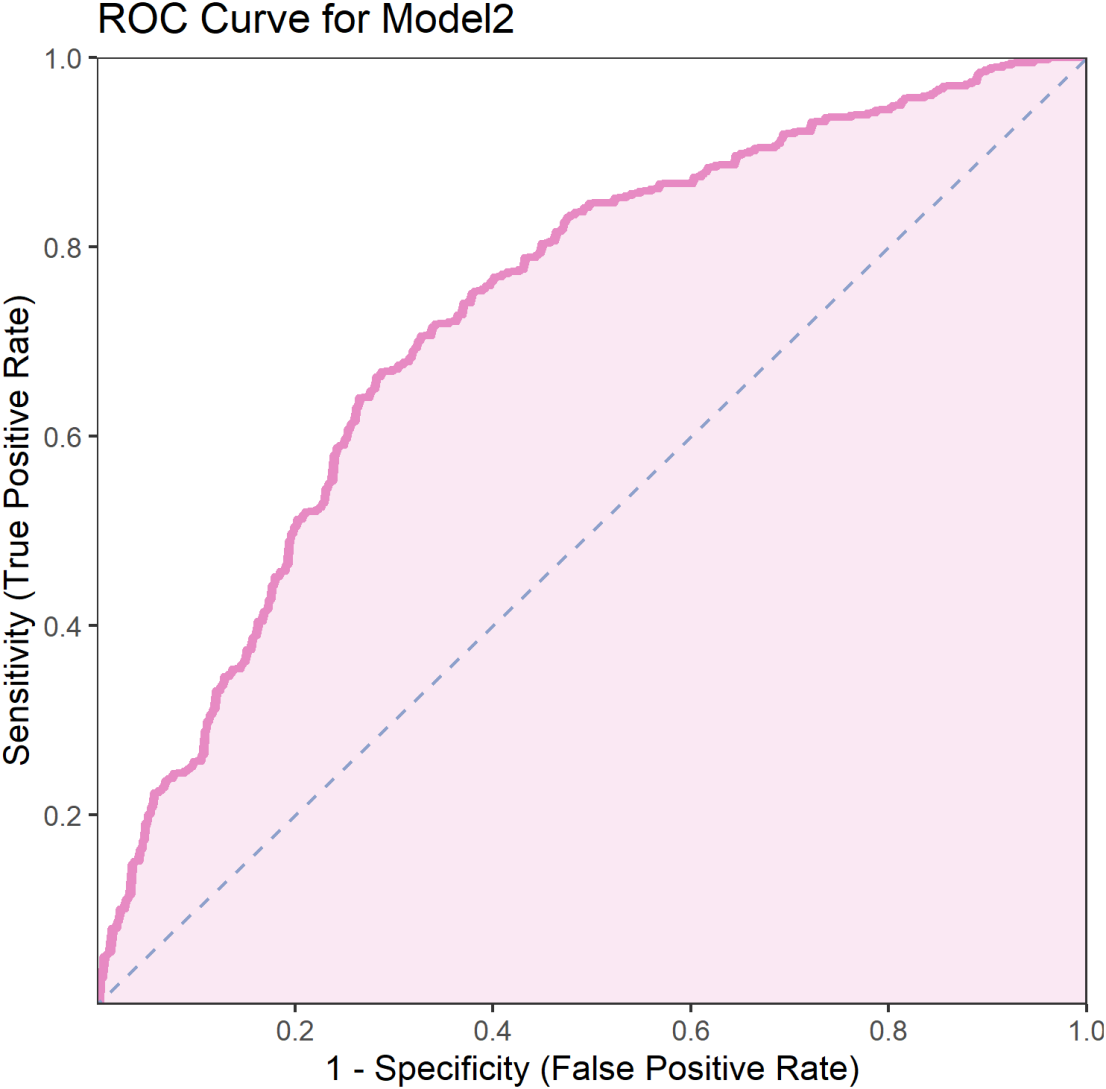
ROC curve for Model 2.

Results showed that model 1 had an AUC of 0.649 (95% CI: 0.610–0.688), with a sensitivity of 0.641, specificity of 0.608, and Youden index of 0.249 (Figure 1). Model 2 had a higher AUC of 0.731 (95% CI: 0.694–0.767), with a sensitivity of 0.663, specificity of 0.719, and Youden index of 0.382 (Figure 2). Overall, model 2, which included the treatment method, outperformed model 1 in predicting asthma control and sleep quality improvement, demonstrating better discrimination ability (Table 6).

**TABLE 6:** ROC curve parameters of two multivariate logistic regression models.

	AUC	AUC‐CI‐Lower	AUC‐CI‐Upper	Threshold	Youden	Sensitivity	Specificity
Model 1	0.649	0.610	0.688	0.540	0.249	0.641	0.608
Model 2	0.731	0.694	0.767	0.529	0.382	0.663	0.719

### 3.7 Stratified Analysis

**FIGURE 3:**
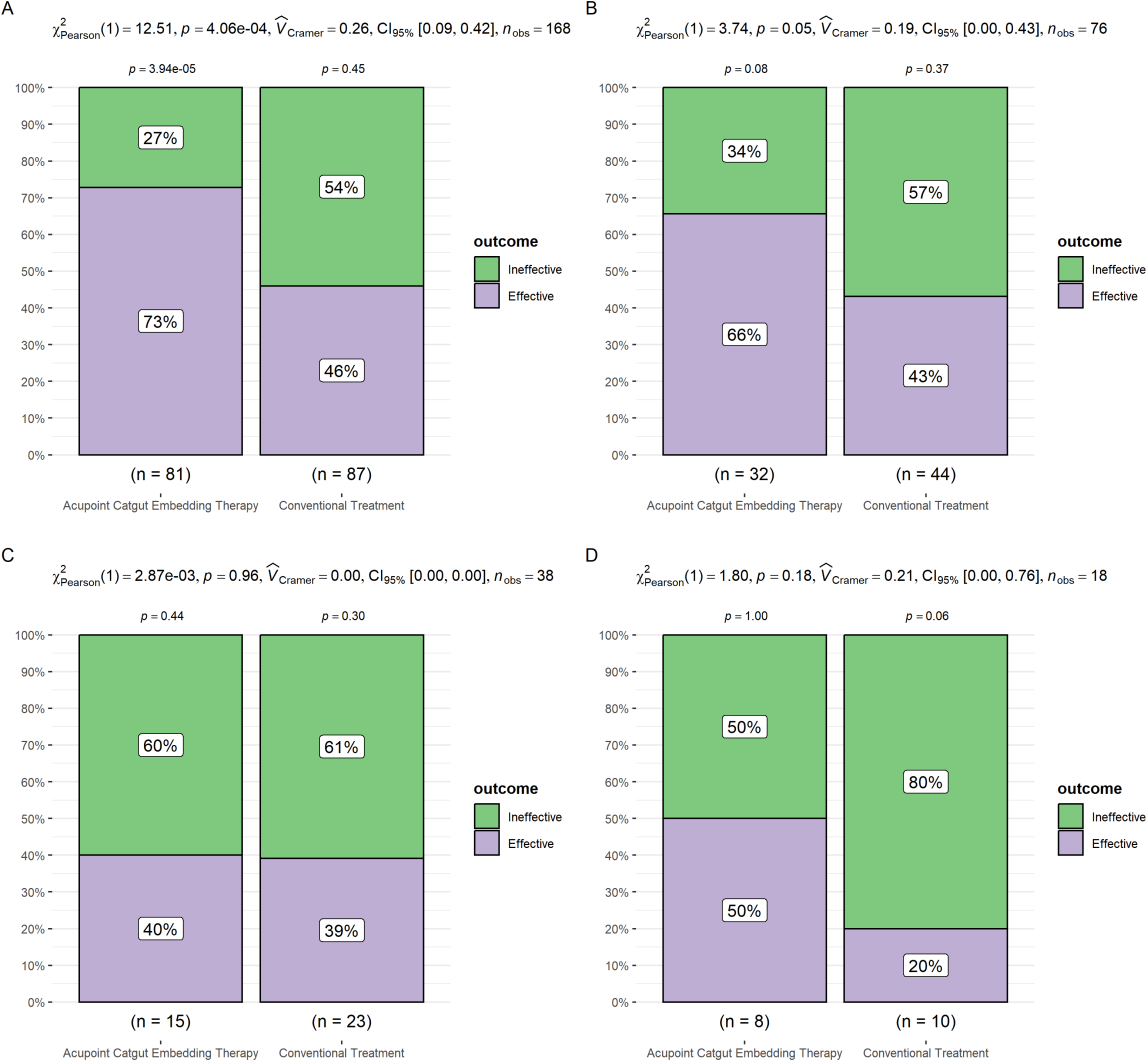
(A) Efficacy comparison between treatment methods in patients with intermittent asthma. (B) Efficacy comparison between treatment methods in patients with mild persistent asthma. (C) Efficacy comparison between treatment methods in patients with moderate persistent asthma. (D) Efficacy comparison between treatment methods in patients with severe persistent asthma.

In patients with intermittent asthma, the effective rate was 73% in the acupoint catgut embedding group and 46% in the conventional treatment group (*p* < 0.001), indicating a highly statistically significant difference between the two groups, with acupoint catgut embedding therapy being significantly more effective than conventional treatment (Figure 3A). In patients with mild persistent asthma, the effective rate was 66% in the acupoint catgut embedding group and 43% in the conventional treatment group (*p* = 0.05750), showing a significant difference, with acupoint catgut embedding therapy being more effective (Figure 3B). In patients with moderate persistent asthma, the effective rate was 40% in the acupoint catgut embedding group and 39% in the conventional treatment group (*p* = 0.96), not statistically significant (Figure 3C). In patients with severe persistent asthma, the effective rate was 50% in the acupoint catgut embedding group and 20% in the conventional treatment group (*p* = 0.18); although the acupoint catgut embedding group had a higher effective rate, the difference was not statistically significant (Figure 3D).

## 4 Discussion

This study, through a retrospective controlled analysis, evaluated the differences in efficacy between acupoint catgut embedding auxiliary therapy and conventional auxiliary treatment in patients with asthma, particularly in terms of asthma control, immune response, sleep quality, and psychological state. The results showed that acupoint catgut embedding therapy significantly outperformed conventional treatment in many aspects, especially in patients with mild‐to‐moderate asthma. These findings not only provide strong evidence for acupoint catgut embedding as an effective treatment option for asthma but also reveal its potential immune and neuroendocrine regulatory mechanisms [17].

First, the study found that the acupoint catgut embedding therapy group significantly outperformed the conventional treatment group in terms of asthma control, frequency of acute exacerbations, anxiety and depression levels, and lung function. This result is consistent with existing traditional Chinese medicine theories. Acupoint catgut embedding therapy may regulate lung function by continuously stimulating specific acupoints, enhancing the contractility of airway smooth muscles, and reducing airway inflammation, thereby improving asthma control. Further multivariate regression analysis confirmed that the treatment method (acupoint catgut embedding) had a significant independent effect on asthma control (*p* < 0.001, OR = 3.693). These findings suggest that acupoint catgut embedding may activate the body's immune regulatory mechanisms, alleviating airway inflammation and achieving better control outcomes.

Secondly, in terms of immunological and inflammatory markers, the levels of IgE, eosinophil count, and IL‐5 in the acupoint catgut embedding group were significantly lower than those in the conventional treatment group, while the IL‐10 levels were significantly higher. IgE (immunoglobulin E) is an antibody belonging to the immunoglobulin family in the human immune system, eosinophils are a type of white blood cell that is part of the immune system [18, 19], and IL‐5 is an important cytokine in the interleukin family [20]. These three markers are closely related to immune‐inflammatory responses. Our study found that acupoint catgut embedding therapy significantly reduced the levels of these three markers, which may be because acupoint catgut embedding stimulates specific acupuncture points, activating related neural reflex pathways, transmitting signals to the central nervous system (CNS), and then influencing immune system functions through the autonomic nervous system, thus inhibiting overly active immune‐inflammatory responses. Acupoint catgut embedding can also improve blood circulation, help clear local inflammatory factors, improve airway function, and alleviate inflammation, thereby reducing the levels of inflammatory markers such as IgE and eosinophils. IL‐10 is one of the most important anti‐inflammatory cytokines, mainly involved in inhibiting immune function and suppressing T‐cell activation. Acupoint catgut embedding therapy, by stimulating specific acupuncture points, activates the sympathetic and parasympathetic nervous systems. Studies have shown that activation of the parasympathetic nervous system is associated with the secretion of IL‐10. As a result, IL‐10 levels increase, suppressing excessive inflammation in the body and controlling asthma symptoms.

In terms of sleep quality, the acupoint catgut embedding group showed significant improvements in PSQI score, number of awakenings, and the proportion of REM and NREM 3 (deep sleep) stages compared to the conventional treatment group. This indicates that acupoint catgut embedding not only improves asthma symptoms but may also optimize sleep structure by regulating the neuroendocrine system, particularly hormones related to sleep, such as melatonin and cortisol [21, 22]. Good sleep can improve patients' quality of life and may reduce airway hyperreactivity, further enhancing asthma control [23]. However, although acupoint catgut embedding showed significant advantages in improving sleep quality, this study did not delve deeply into its effects on specific neuroendocrine mechanisms, which is an important direction for future research.

Compared with model 1, model 2, which included the auxiliary treatment method variable, had a significantly higher AUC value (increased from 0.649 in model 1 to 0.731 in model 2). This indicates that the contribution of the treatment method to improving asthma control and sleep quality is independent and strong. In other words, acupoint catgut embedding therapy had an independent and significant positive effect on asthma control and sleep quality, which was not fully reflected in model 1 without the treatment method variable. The inclusion of the treatment method allowed the model to more accurately differentiate patients with good asthma control and improved sleep quality from others. Thus, model 2 was able to capture the impact of various variables on asthma control and sleep quality more comprehensively, further proving that the addition of the treatment method significantly enhanced the model's discriminative ability. This suggests that incorporating acupoint catgut embedding therapy into comprehensive asthma management could be an important strategy for improving treatment outcomes. Compared to conventional treatment, acupoint catgut embedding can provide more health benefits for patients.

Furthermore, stratified analysis revealed that acupoint catgut embedding therapy was significantly more effective than conventional treatment in patients with intermittent, mild persistent, and moderate persistent asthma, while there was no statistically significant difference between the two in patients with severe persistent asthma. This could be related to the pathological characteristics of severe asthma, as these patients typically exhibit more complex airway remodeling and stubborn airway inflammation [24]. A single peripheral stimulus, such as acupoint catgut embedding, may not achieve significant effects in such cases. Therefore, for patients with severe asthma, it may be necessary to combine other treatment methods, such as stronger anti‐inflammatory drugs or biological agents, to achieve better treatment outcomes.

Although acupuncture point embedding therapy has good therapeutic effects, it also carries potential side effects. Common adverse reactions include localized pain, redness, swelling, itching, bruising, and temporary discomfort, which usually subside after a period of time. There have also been reports of infection and allergic reactions, although these are rare. This highlights the importance of strict aseptic techniques during acupuncture point embedding therapy to minimize the occurrence of such side effects.

Since this study was conducted during the COVID‐19 pandemic, it may have had an impact on the study results. During the pandemic, patients' psychological state could have been influenced by the dual impact of asthma and COVID‐19, leading to an exacerbation of anxiety and stress. Anxiety and stress could increase the body's inflammatory response, indirectly worsening airway inflammation, thus aggravating asthma symptoms and leading to poorer outcomes for all treatment methods. Future studies could include a control group from a non‐pandemic period to analyze the effect of COVID‐19 on treatment efficacy.

This study also has certain limitations. First, as a retrospective study, selection bias cannot be completely avoided. Second, this study only assessed immune responses using a limited range of biomarkers, so future studies should introduce a broader range of immunological indicators to more comprehensively understand the mechanisms of acupoint catgut embedding therapy. In this study, we only collected data at a single time point, 12 weeks after treatment, providing preliminary efficacy data. Future studies should include a longer follow‐up period and collect patient data at multiple time points to observe the sustained effects of acupoint catgut embedding therapy on immune‐inflammatory responses and clinical benefits.

## 5 Conclusion

Overall, acupoint catgut embedding therapy can improve asthma symptoms, enhance lung function, and improve sleep quality by regulating immune‐inflammatory responses. It is more effective in patients with mild‐to‐moderate asthma and can serve as an important option for future asthma treatment and management.

## Author Contributions

Zhihong Shi was responsible for study conception and design, data analysis, and manuscript drafting. Wulanqiqige contributed to data extraction and interpretation from medical records. Qing Quan and Narentuya Zhao participated in literature review and statistical analysis. Yanan Wang and Xiaohong Bai assisted in data collection and verification. Xiaoyan Hu supervised the study and critically revised the manuscript. All authors read and approved the final manuscript.

## Ethics Statement

This study is a retrospective and anonymized study. All patient data used were obtained from existing clinical records, and patient privacy and confidentiality were strictly protected during data processing. The study protocol was approved by the Ethics Committee of the Mongolian Medical Department, Affiliated Hospital of Inner Mongolia Medical University (Approval Number: IMMU‐2024‐B21). Given that the study utilized anonymized data and involved no additional interventions, the Ethics Committee granted a waiver of written informed consent from patients.

## Conflicts of Interest

The authors declare no conflicts of interest.

## Data Availability

The data that support the findings of this study are available from the corresponding author upon reasonable request.
